# Noninvasive prenatal detection of fetal sex chromosome abnormalities using the semiconductor sequencing platform (SSP) in Southern China

**DOI:** 10.1007/s10815-020-02056-2

**Published:** 2021-02-10

**Authors:** Jiexia Yang, Yaping Hou, Fangfang Guo, Haishan Peng, Dongmei Wang, Yi Li, Haoxin OY, Yixia Wang, Jian Lu, Aihua Yin

**Affiliations:** 1 Department of Medical Genetics Center, Guangdong Women and Children Hospital, Guangzhou, China; 2 Department of Prenatal Diagnosis Center, Guangdong Women and Children Hospital, No. 521 Xingnan Road, Panyu District, Guangzhou, 511400 China

**Keywords:** Noninvasive prenatal test (NIPT), Sex chromosome aneuploidies (SCAs), Positive predictive values (PPVs), Copy number variations (CNVs), Mosaicism

## Abstract

**Background:**

Noninvasive prenatal testing (NIPT) has been widely used to screen for fetal aneuploidies, including fetal sex chromosome aneuploidies (SCAs). However, there is less information on the performance of NIPT in detecting SCAs.

**Methods:**

A cohort of 47,800 pregnancies was recruited to review the high-risk NIPT results for SCAs. Cell-free fetal DNA (cffDNA) was extracted and sequenced. All NIPT high-risk cases were recommended to undergo invasive prenatal diagnosis for karyotyping analysis and chromosome microarray analysis (CMA).

**Results:**

A total of 238 high-risk cases were detected by NIPT, including 137 cases of 45,X, 27 cases of 47,XXX, and 74 cases of 47,XYY/47,XXY. Prenatal diagnosis, including karyotyping analysis and CMA, was available in 170 cases. The positive predictive value (PPV) was 30.00% for 45,X, 70.58% for 47,XXX, and 81.13% for 47,XYY/47,XXY. In addition, 13 cases of sex chromosome mosaicism and 9 cases of sex chromosome CNVs were incidentally found in this study.

**Conclusion:**

Our study showed that NIPT was reliable for screening SCAs based on a large sample, and it performed better in predicting sex chromosome trisomies than monosomy X. Our study will provide an important reference for clinical genetic counseling and further processing of the results.

## Introduction

Sex chromosome aneuploidies (SCAs) are numeric abnormalities of sex chromosomes that are relatively common genetic conditions, affecting as many as 1/400 newborns, approximately twice as frequent in newborns as trisomy 21 [1]. The most common SCA karyotypes include 45,X (Turner syndrome), 47,XXY, 47,XXX (Triple X syndrome), 47,XYY syndrome, and sex chromosome mosaicism. The most common symptoms are not life-threatening and are associated with highly variable degrees of clinical problems in the physical, reproductive, and behavioral domains, and the clinical prognosis is relatively good [2–4]. Therefore, early intervention and proper postnatal management would improve the quality of life of the affected children [3].

Noninvasive prenatal testing (NIPT) has been widely used to screen pregnant women for fetal aneuploidies, including fetal SCAs [5, 6]. Predictably, NIPT has become a first-tier screening for aneuploidies in low- and high-risk populations, and there will likely be a significant increase in the incidence of positive screening results for SCAs [7]. However, there is still limited information about the ability of NIPT in detecting SCAs. At present, there are two main problems with SCA testing: (1) the small sample size of the cohorts and extensive loss to follow-up, making evaluation of NIPT efficiency in detecting SCAs and parental choice limited; and (2) most of the laboratories offering NIPT include sex chromosome testing as a part of this test, since prospective parents are eager for detailed information.

In the present study, we focused on the overall performance of NIPT in screening SCAs and prenatal decision-making in clinical practice with a large cohort and provided an important reference for clinical genetic counseling and further processing of the results.

## Materials and methods

### Participant recruitment

This retrospective study enrolled pregnant women who attended Guangdong Women and Children Hospital and underwent NIPT detection from January 2015 to September 2019. The inclusion criteria were as follows: (i) pregnant women aged 18–45 years and (ii) gestational age greater than 12 weeks. Gestational age was determined by ultrasound. The study was approved by the Institutional Review Board of Guangdong Women and Children Hospital, and each participant had detailed genetic counseling and signed written informed consent prior to participation.

### Sample preparation and sequencing

Peripheral blood samples (5–10 ml) were withdrawn from the cubical veins of pregnant women, which were collected in EDTA within 8 h, or cell-free DNA was collected in BCT tubes (Streck Inc.; Omaha, NE) within 72 h at 4 °C. Samples were processed, and plasma was shipped frozen when necessary. Trisomies 13, 18, and 21 were reported as well as other chromosomal aneuploidies. Cell-free DNA extraction, library construction, sequencing, and bioinformatics analysis were performed according to a previous study. High-throughput sequencing of fetal-free DNA fragments was performed using a JingXin BioelectronSeq 4000 System (CFDA registration permit No. 20153400309) semiconductor sequencer. Sequencing reads were filtered and aligned to the human reference genome (hg19) [8]. The combined GC correction and *Z* score testing methods were used to identify fetal autosomal aneuploidy. Each chromosome with an absolute *Z* score greater than 3 was marked with chromosome aneuploidies or microdeletions/microduplications; see our previous article for details [9].

### Prenatal diagnosis and pregnancy follow-up

Pregnant women who had a high risk of NIPT results underwent genetic counseling and were fully informed about undergoing prenatal diagnosis. Chromosomal detection techniques include karyotyping (the resolution of G-banding was 400 bands) and chromosome microarray analysis (CMA) (CytoScanTM 750 K, available from Affymetrix, USA). To obtain information about neonatal outcomes and newborn growth, we followed up all participants via telephone interviews.

### Chromosome karyotype analysis

Chromosome karyotype analysis under sterile conditions was performed on fetal DNA, cultured amniocytes, and lymphocytes according to standard protocols. The amniotic fluid was centrifuged, the precipitated cells were collected, and the cells were cultured in situ. After G-banding, each slide was observed under a microscope, and 20 to 30 cells were counted and analyzed with special software for chromosome analysis.

### CMA

Fetal genomic DNA was extracted from amniotic fluid or cord blood using a QIAamp DNA Blood Mini Kit (Qiagen, Germany). The DNA (300 ng) was amplified, labeled, and hybridized by using the CytoScan 750 K array platform (Affymetrix, USA) according to the manufacturer’s protocol. Data were visualized by scanning with CytoScan^TM^ and analyzed with Chromosome Analysis Suite software (Affymetrix, USA) based on the GRCH37 (hg19) assembly.

## Results

### Patient characteristics

**Fig. 1:**
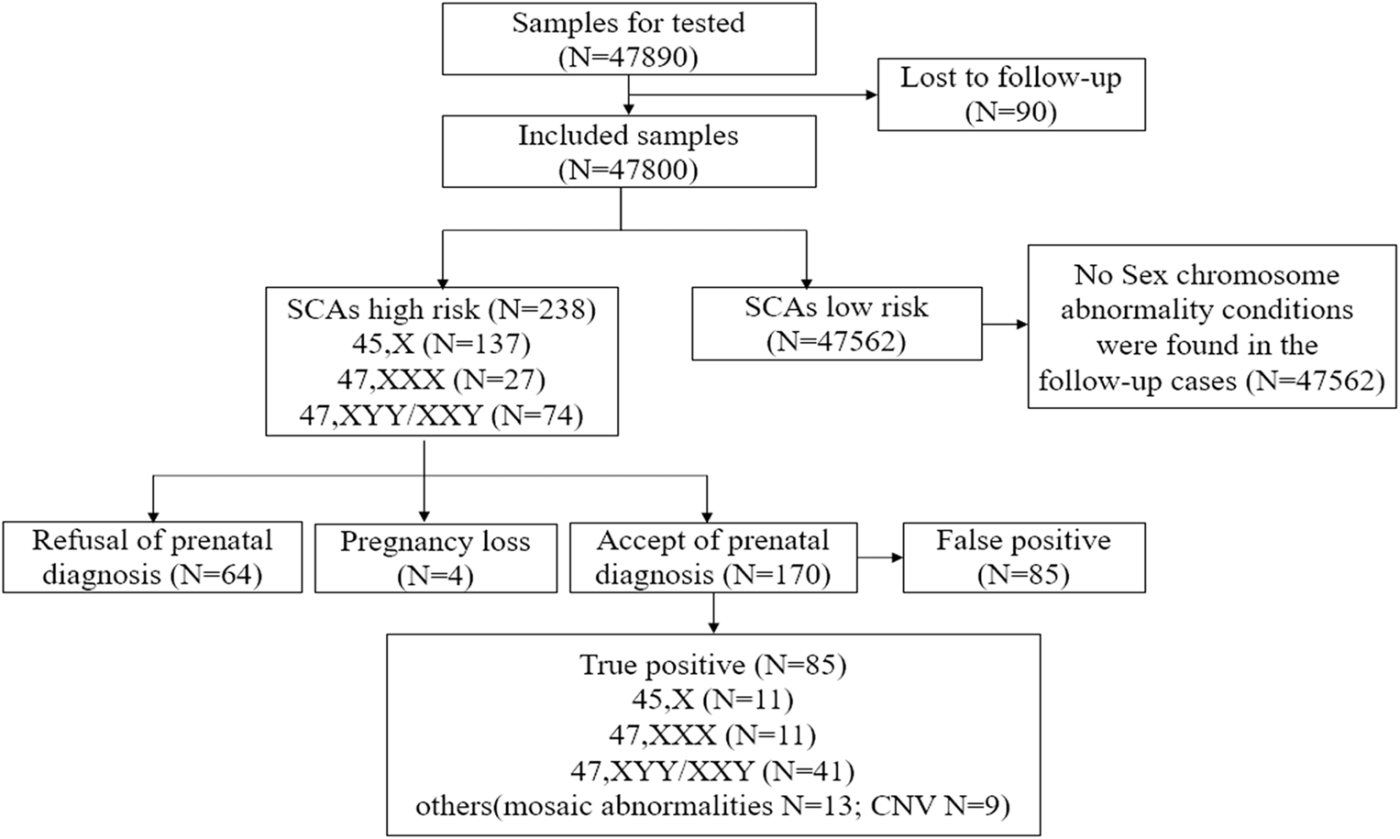
Flowchart of noninvasive prenatal test (NIPT) results and the clinical outcomes of pregnant women

From January 2015 to September 2019, 47,890 cases were enrolled for NIPT detection. Ninety cases were excluded due to loss of follow-up, resulting in a total of 47,800 cases being included in this study. The majority (80.8%) of pregnant women were at 12–24 gestational age when NIPT was performed, and 29.03% were at high risk for serological screening. Furthermore, 5690 were advanced maternal age women (age ≥ 35 years), and 1530 were twin pregnancies. There were 2629 in vitro fertilization (IVF) pregnancies (Table 1 and Fig. 1).

**Table 1:** Demographic characteristics of the 47,800 pregnancies examined by NIPT

Characteristic	Number (%)
Total	47,800 (100.00%)
Singleton pregnancy	46,270 (96.8%)
Twin pregnancies	1530 (3.2%)
Gestational age at NIPT	
12–19^+6^ weeks	25782 (53.94%)
20–23^+6^ weeks	12838 (26.86%)
24–29^+6^ weeks	5618 (11.75%)
30–34^+6^ weeks	3298 (6.9%)
≥ 35 weeks	264 (0.55%)
Routine prenatal screening results	
High risk	13875 (29.03%)
Intermediate risk	18342 (38.37%)
Low risk	10290 (21.53%)
Ultrasound soft index abnormalities	5293 (11.07%)
Maternal age, years	
< 35	42110 (88.10%)
35–39	5306 (11.10%)
≥ 40	384 (0.80%)
IVF pregnancies	2629 (5.9%)
Fetal fragment fraction	13.11% (CI: 5.53−17.70%)

### NIPT results

A total of 238 cases were predicted to have a high risk of SCAs by NIPT in 47800 samples; among them, 170 cases accepted prenatal diagnosis, 64 cases refused further prenatal diagnosis, and 4 cases aborted in the second trimester.

### Interventional prenatal diagnosis results

All 238 high-risk cases predicted by NIPT were fully informed about undergoing prenatal diagnosis; 170 cases accepted prenatal diagnosis, and 64 cases refused. This confirmed 85 true-positive and 85 false-positive cases of fetal sex chromosomal abnormalities. Additionally, the positive predictive values (PPVs) for each test were assessed. The PPV of NIPT for 45,X was 30.00%; for 47,XXX, it was 70.58%; and for 47,XYY/47,XXY, it was 81.13%. Our data showed that NIPT performed better in predicting sex chromosome trisomies than monosomy X. In addition, another 13 cases of fetal sex chromosome mosaicism and 9 cases of CNV were found incidentally (Table 2).

**Table 2:** Performance of NIPT screening for fetal SCAs in patients who had undergone prenatal diagnosis

NIPT predicted SCA type	Prenatal diagnostic validated	True positive		False positive	PPV (%)	Without validated
		Karyotype	Number			
45,X	100	45,X	11	70	30/100 (30.00%)	38
		mos 45,X/46,XX	11			
		CNV	8			
47,XXX	17	47,XXX	11	5	12/17 (70.58%)	20
		mos 47,XXX/46,XX	1			
47,XYY/47,XXY	53	47,XYY	13	10	43/53 (81.13%)	10
		47,XXY	28			
		mos 47,XYY/47,XY	1			
		CNV	1			
Total	170	/	85	85	85/170 (50.00%)	68

*PPV* positive predictive value

### Detection of sex chromosome mosaicism

**Fig. 2:**
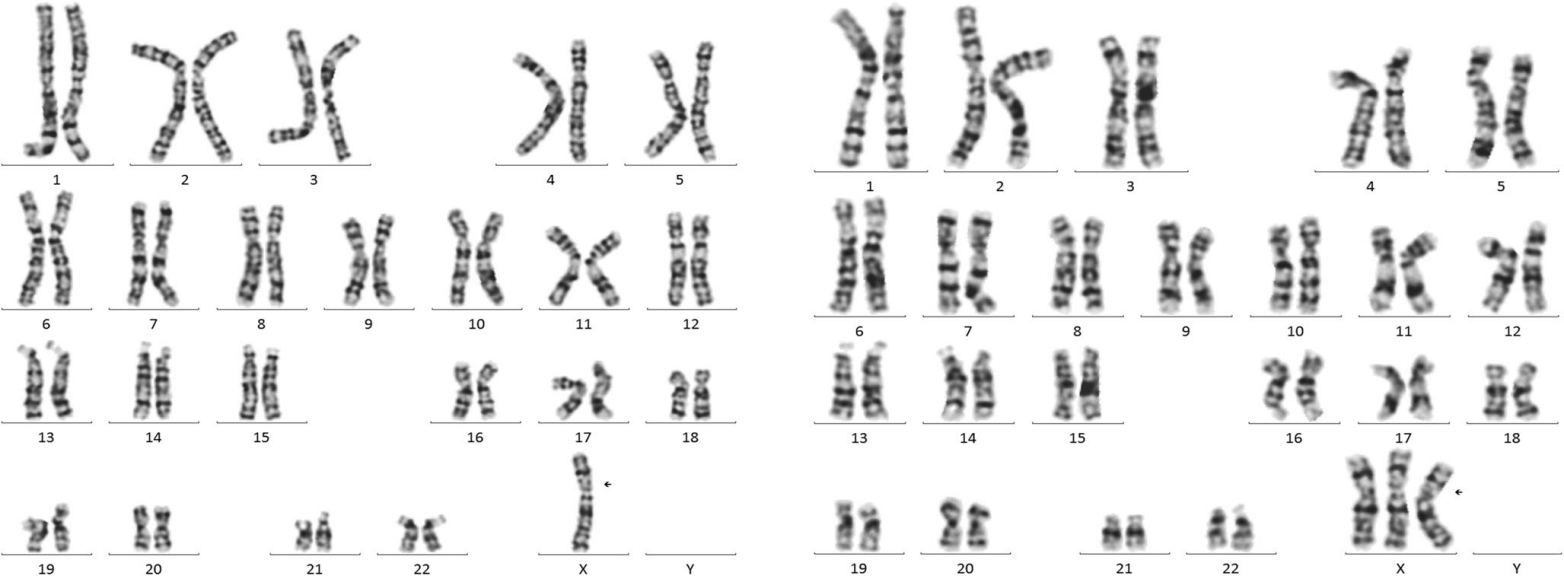
Karyotype analysis of the peripheral blood of pregnant women in case 8. The arrow indicates that the mother’s karyotype is a mosaic of X chromosome monosomy and X chromosome trisomy

In the 170 high-risk patients who underwent prenatal diagnosis, 13 mosaicism cases were confirmed (Table 3). For all mosaicism cases, the fetal fragment fraction was 7.9–24.1%. The *Z* score of the X chromosome in NIPT was − 3.83 to − 8.94. The prenatal diagnosis results confirmed different degrees of chimerism of the fetal sex chromosomes except in case 8. It is worth mentioning that case 8 was suspected to have X/XY mosaicism by NIPT, but it was confirmed to be false positive by prenatal diagnosis and karyotype analysis of the peripheral blood of the pregnant woman (Fig. 2). The fetal chromosome karyotype was normal, and the mother’s chromosome karyotype was mos 45,X[85]/47,XXX[15]. Thus, the false-positive NIPT results were due to a background of maternal chromosome abnormalities. Cases 3, 7, 10, 11, and 13 chose to terminate the pregnancy. The other cases chose to continue the pregnancy, and we learned through follow-up that these children are now developing normally.

**Table 3:** Detection of sex chromosome mosaicism

Case	NIPT results	Results of prenatal diagnosis	Proportion of mosaic anomalies	Maternal age (years)	*Z* score	cffDNA concentration	Maternal karyotype
Case 1	45,X	mos45,XO[7]/46,XX[23]	23.33%	27	− 3.83	16.4	46,XX
Case 2	45,X	mos45, X[2]/46,XX[18]	10%	34	− 4.91	7.9	46,XX
Case 3	45,X	mos 45,X[22]/46,X,+mar[4]	84.62%	30	− 8.94	14.9	46,XX
Case 4	45,X	mos45,X[7]/46,XX[11]	38.89%	30	− 8.44	13.6	46,XX
Case 5	45,X	mos45,XO[23]/46,XX[12]	65.71%	29	− 7.50	13.3	46,XX
Case 6	45,X	mos45,XO[3]/46,XX[18]	66.67%	29	− 6.25	17.7	46,XX
Case 7	45,X	mos45,XO[29]/46,XX[7]	85%	34	− 6.62	9.3	46,XX
Case 8	45,X/46,XY	46,XY	0	29	− 0.80	24.1	mos 45,X[85]/47,XXX[15]
Case 9	45,X	mos 45,X[3]/46,XX[32]	8.60%	29	− 7.31	14.1	46,XX
Case 10	45,X	mos45,XO[27]/46,XX[10]	73%	35	− 7.48	17.4	46,XX
Case 11	45,X	mos45,XO[37]/47,XXX[8]	-	35	− 7.81	17.419	46,XX
Case 12	47,XXY	46,X,der(Y;Y)(q11.22;q11.22)del(Y)(p11.32)[97]/45,X[3]	-	26	− 1.69	10.245	-
Case 13	47,XXX	46,XX[53]/47.XXX[47]	47%	31	− 5.84	19.367	46,XX

### Detection of sex chromosome copy number variations

**Fig. 3:**
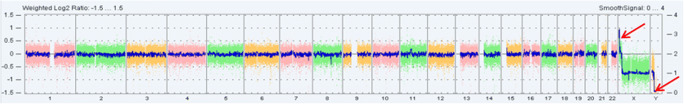
CMA results of case 18. The arrow indicates that the Y chromosome had a 13.7 Mb deletion and the X chromosome had a 4.1 Mb duplication

**Fig. 4:**
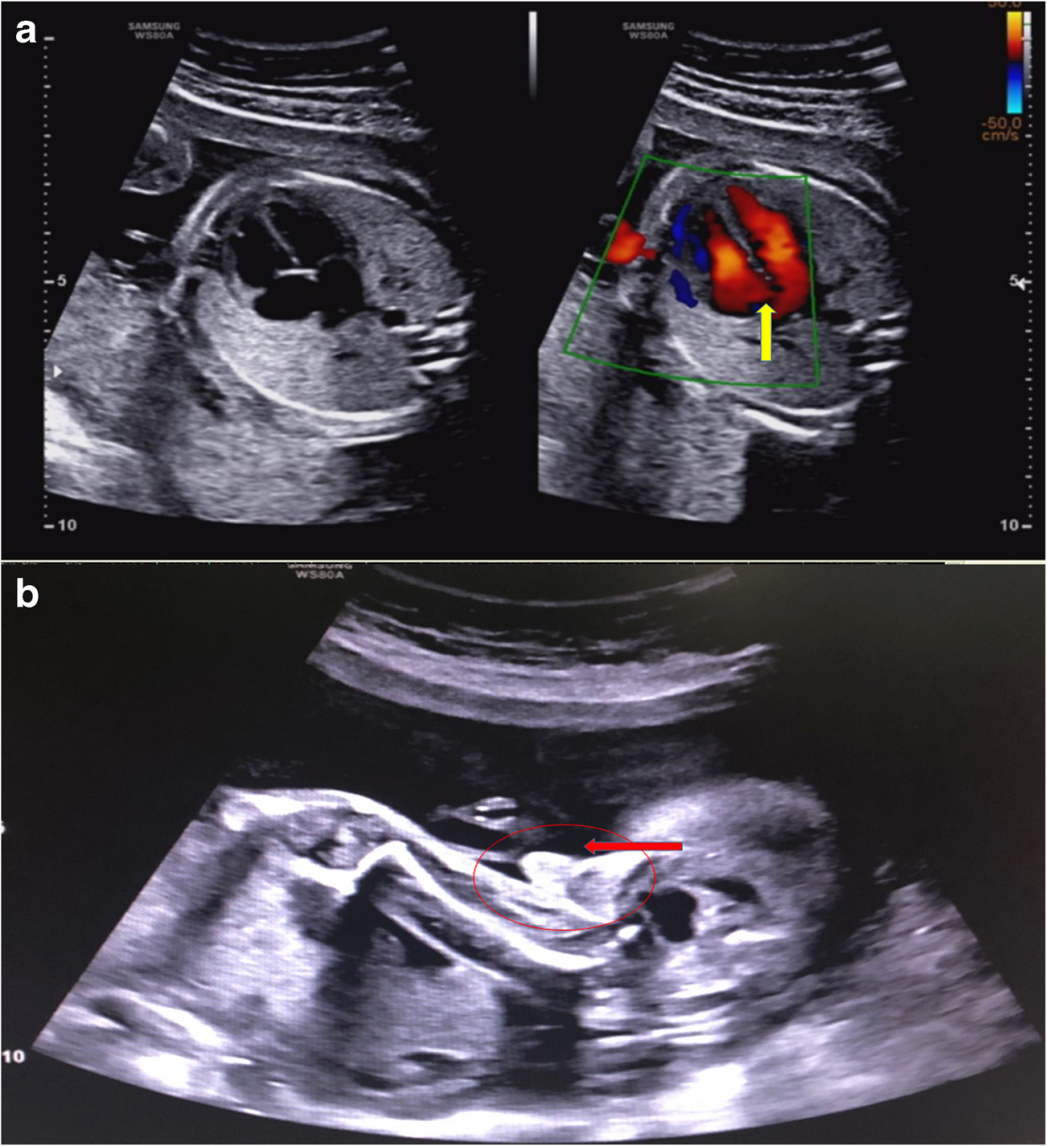
Ultrasound examination results of case 18. (**a**) Ultrasound results suggest tricuspid regurgitation. (**b**) The maleness of the fetus was confirmed at 24 weeks

In addition, prenatal diagnosis confirmed 9 copy number variation (CNV) cases (Table 4). For all CNV cases, the fetal fragment fraction was 10.6–28.8%. The *Z* score of the X chromosome in NIPT was − 2.562 to − 17.657. Interestingly, case 18 was suspected to have X/XY mosaicism by NIPT, and CMA confirmed the large segments of deletion and duplication of the sex chromosome, including the Y chromosome, which had a 13.7 Mb deletion and the X chromosome had a 4.1 Mb duplication (Fig. 3). The fetus was confirmed as male at 24 weeks by ultrasound examination and the ultrasound results also suggested tricuspid regurgitation (Fig. 4). All of the CNV cases chose to terminate the pregnancy. Case 21 chose to terminate the pregnancy and agreed to a fetal autopsy. The autopsy results confirmed tissue degeneration.

**Table 4:** Detection of sex chromosome copies number variation

Case	NIPT results	Results of prenatal diagnosis	Maternal age (years)	*Z* score	cffDNA concentration
Case 14	45,X	46, X,der(X)t(X;?)(P22.3;?)	39	− 8.577	10.6
Case 15	45,X	arr[hg19]Xp22.33 or Yp11.32(168,551-1,623,390 or 118,551-1,573,390)×1	27	− 6.925	11.1
Case 16	45,X	arr[hg19]Xp22.33-q26.2(0-130,940.381)×1-2/Xq26.2q28(130,940,382-155,233,098)×1	28	− 8.194	14.6
Case 17	45,X	arr[hg19]Xp22.3p11.4(168,551-39,696,595)×1 /Xp11.4q25(39,722,729-123,397,520)×1-2/Xq25q28(123,485,861-155,233,098)×1	29	− 15.05	28.8
Case 18	45,X/46,XY	arr[hg19]Xp22.33p22.2(168,551-14,253,462)×2	28	− 17.657	10.5
		Yp22.33p22.2(15,016,171-28,712,930))×0			
Case 19	45,X	arr(Xp)×1,(Xq)×1-2	26	− 7.047	14.3
Case 20	45,X	arr[hg19]Xp22.33p11.1(168,551-58,527,155)×1	31	− 16.011	16.3
		Xq11.1q21.1(61,957,811-77,549,045)×1-2			
		Xq21.1q28(77,624,194-155,233,098)×1			
Case 21	45,X	arr[hg19]Xp22.33p21.3(168,551-28,291,878)×1	34	− 2.562	18.9
Case 22	45,X	arr[hg19]Xp22.33-q26.2(0-130,940.381)×1	25	− 7.05	13.3

### NIPT performance for screening fetal SCAs according to the pregnancy characteristics

In this study, in addition to NIPT testing, the pregnant women also underwent other prenatal screening, including serological screening and ultrasound examinations. Thus, pregnancies could be divided into different characteristics according to these screening results. We also analyzed the NIPT performance according to different pregnancy characteristics. Pregnant women with a high risk of serological screening had the highest positive predictive value (53.03%), and pregnant women with abnormal ultrasound findings had the lowest positive predictive value (37.50%) (Table 5). In addition, NIPT predicted 9 cases of SCA high-risk pregnancies among 1530 twin pregnancies. Only 6 high-risk pregnancies underwent prenatal diagnosis, which confirmed 3 cases of true positives and 3 cases of false positives, resulting in a PPV for SCA in twin pregnancies of 50.00%.

**Table 5:** Performance of NIPT for screening for fetal SCAs according to pregnancies’ characteristics

Characteristic	NIPT high risk of SCAs	Prenatal diagnostic validated		PPV(%)
		True positive	False positive	
High risk of serological screening	75	35	31	53.03% (35/66)
Intermediate risk of serological screening	86	28	28	50.00% (28/56)
Low risk of serological screening	30	9	11	45.00% (9/20)
Abnormal ultrasound findings	12	3	5	37.50% (3/8)
Advanced maternal age	35	10	10	50.00% (10/20)
Total	238	85	85	50.00% (50/100)

### Follow-up low-risk pregnancies and pregnancies who declined prenatal diagnosis

At the time of writing, all pregnant women included in this cohort had given birth. To date, no false negative results have been found among the 47,562 NIPT low-risk pregnancies, and 64 patients who refused prenatal diagnosis were also followed up. All pregnant women had given birth successfully. No visible abnormalities were found in the newborn screening. However, given the special nature of sex chromosomal abnormalities, these cases need to be followed up for a long time.

## Discussion

NIPT has been widely used to screen for trisomy 21, trisomy 18, and trisomy 13 for many years. Subsequently, the technology has advanced such that NIPT is available for screening for SCAs. However, NIPT was inferior in the prediction of SCAs to trisomies 21, 18, and 13. According to our data, 238 high-risk cases of SCAs among 47,800 pregnant women were found, and the majority of these women (71.42%, 170/238) chose to undergo invasive testing, which agreed with data reported before [10, 11]. The overall PPV for fetal SCAs was 50.00%. When categorized by individual SCA, the PPV was 30.00% for 45,X, 70.58.00% for 47,XXX, and 81.13.00% for 47,XYY/47,XXY, which was similar to previous reports [7, 12, 13]. Our findings demonstrated that NIPT performed better in predicting sex chromosome trisomies than monosomy X, which agreed with Xu’s report [5]. Many reports have shown that the PPV for 45,X ranges from 20 to 30% [7, 14].

However, there were also some discordant situations. Thus, the NIPT results were discordant with the prenatal diagnosis in some cases. Possible explanations include the following: (1) maternal SCA (full or mosaic) caused a discordant SCA, such as case 8, which is a common and important reason. Wang’s article reported that 8.6% of positive cffDNA results for SCA were due to maternal sex chromosome mosaicism [15]. (2) Another reason is confined placental mosaicism (CPM), which occurs in approximately 1% of pregnancies [16]. NIPT used cell-free fetal DNA (cffDNA) for testing, and the primary source of cffDNA is thought to be the apoptosis of placental cells from the cytotrophoblast [17]. There is a situation in which a chromosomal abnormality that occurs only in the placenta but not in the fetus is known as CPM. Therefore, collecting placental tissue for further verification is a critical part of confirming CPM, which is reported in Liu’s latest article [18]. In this study, no placental tissue was obtained for verification, which was a limitation of the present study.

In addition, 9 cases of sex chromosome CNVs were found, but the NIPT results showed sex chromosome aneuploidy. Case 15 was suspected to be X/XY mosaicism by NIPT, but CMA by amniocentesis confirmed that the Y chromosome had a 13.7-Mb deletion and the X chromosome had a 4.1-Mb duplication. Sexual chromosomal abnormalities detected by NIPT were caused by changes in the ratio of fetal X to Y chromosomes, which was also reported in a previous study [19]. Reviewing the pregnancy outcomes, we found that all sex chromosome CNV cases chose to terminate the pregnancy. A previous study showed that the termination rate for fetal SCAs was 96% in 2008 [20] and 84% in 2013 [21] in mainland China. Differences in parental decisions may be associated with the types of SCAs, maternal age, history of infertility, and even family support, financial support, social support, medical support, and parental and social acceptance of disability [22].

In addition, NIPT showed a better performance in detecting SCAs in the groups with a high risk of serological screening, intermediate risk of serological screening, and advanced maternal age in this study. In a previous study, Chen also analyzed the PPVs according to the pregnancy characteristics. Our data showed a higher PPV compared to Chen’s study [23]. In the present study, the PPV of the low-risk serological screening group was lower than that of the high-risk, intermediate-risk, and advanced-age groups. However, NIPT was also effective for fetal sex chromosome aneuploidy screening. In a recent study, the authors confirmed that NIPT screening for women with low- and critical-risk pregnancies as determined by serological screening could significantly improve the rate and accuracy of fetal chromosomal abnormality detection [24]. Serological screening has a high false-positive rate; thus, NIPT used in high serological screening risk pregnancies may reduce the number of invasive prenatal diagnosis procedures [18]. One point to mention is that the PPV for abnormal ultrasound findings was lower than that of the other groups. Abnormal ultrasound findings included short long bones (SLBs), a thickened nuchal fold, an echogenic intracardiac focus, an absent or hypoplastic nasal bone, and hyperechogenic bowels, which were more commonly seen as autosomal aneuploidies, such as trisomy 21, trisomy 18, and trisomy 13. Perhaps this is the main reason for the PPV of SCAs in abnormal ultrasound findings being lower.

Detection of SCAs in routine prenatal diagnosis is often incidental and presents unforeseen findings to the parents. There are still some issues that require further consideration. For example, some SCAs are without consequences but probably lead to terminations of pregnancy, an increase in the rate of sex selection [10], and increased difficulty in genetic counseling and additional parental anxiety [25]. However, there are benefits of screening for SCA by NIPT. One study showed positive effects of postnatal hormone therapy on the behavioral phenotype if applied earlier to SCA patients [26], and most experts favor an incidental prenatal diagnosis of SCAs [27].

NIPT is a screening test. The American College of Medical Genetics and Genomics (ACMG), the American College of Obstetricians and Gynecologists (ACOG), and the Society for Maternal Fetal Medicine (SMFM) recommend that all pregnant women be informed about the availability of NIPT for SCAs. However, they also emphasized the importance of confirming any positive NIPT results via diagnostic testing. Furthermore, it is necessary to verify the accuracy of NIPT screening for SCAs in large multicenter samples.

## Conclusion

In conclusion, this study obtained a more reliable PPV for screening SCAs based on a large sample that underwent NIPT, and it has provided an important reference for clinical genetic counseling and further processing of results. A limitation was that placental testing was not performed for all false-positive cases; therefore, confined placental mosaicism could not be ruled out. The accuracy of NIPT screening for SCAs needs to be verified by large multicenter samples.

## Data Availability

The datasets used and/or analyzed during the current study are available from the corresponding author on reasonable request.

## Abbreviations

NIPT: noninvasive prenatal testing
SCA: sex chromosome aneuploidies
CMA: chromosome microarray analysis
IVF: in vitro fertilization
PPV: positive predictive values
CNV: copy number variations
cffDNA: cell-free fetal DNA
CPM: confined placental mosaicism
SLB: short long bones
ACMG: American College of Medical Genetics and Genomics
ACOG: American College of Obstetricians and Gynecologists
SMFM: Society for Maternal Fetal Medicine
TOP: termination of pregnancy
